# Prediction and layout optimization of older adult(s) care facilities based on POI data, machine learning, and space syntax

**DOI:** 10.3389/fpubh.2026.1875976

**Published:** 2026-07-22

**Authors:** Yalun Lei, Jingwen Tian, Chuan Wang, Jia Hu

**Affiliations:** 1 School of Art and Design, Shanghai Polytechnic University, Shanghai, China; 2 College of Design and Innovation, Tongji University, Shanghai, China; 3 College of Design and Information, Jiaxing Nanyang Polytechnic Institute, Jiaxing, China; 4 College of Art and Creativity, Anhui University of Applied Technology, Hefei, China

**Keywords:** older adult(s) care facilities, machine learning, POI data, site selection, space syntax

## Abstract

**Introduction:**

As the population continues to age, the scientific selection and rational layout of older adult(s) care facilities are increasingly becoming key issues in urban planning. Yet, traditional planning methods have clear limitations in terms of spatial precision and practicality. This study focuses on Shanghai, a megacity experiencing rapid population aging. It explores a three-tiered, progressive framework for predicting the location of older adult(s) care facilities that integrates POI data, machine learning algorithms, and space syntax.

**Method:**

Shanghai was divided into 33,010 500 × 500-m grid cells, and a training dataset was constructed using POI data. The C5.0 decision tree algorithm was employed, and a multi-algorithm benchmark comparison was conducted with logistic regression and random forests. Through a three-tiered progressive screening process—comprising citywide grid prediction, secondary screening based on aging data, and space syntax-based local choice verification—the study sequentially identifies preliminary suitable grids, priority deployment grids, and key planning grids.

**Results:**

The study found that: (1) The C5.0 decision tree model achieved a prediction accuracy of 83.36%, outperforming logistic regression (82.05%) and random forests (80.96%), indicating that the method is robust and reliable. The model identified 940 preliminarily suitable grid cells, with a spatial match rate of 89% compared to existing older adult(s) care facilities (χ^2^ = 30,517, *p* < 0.001). Catering, real estate, and company facilities were identified as key influencing factors, indicating that existing older adult(s) care facilities tend to cluster in areas with convenient daily services, high residential density, and accessible employment opportunities. (2) Based on the aging rates and older adult population sizes of each subdistrict, a secondary screening was conducted to identify 519 priority deployment grids. These areas exhibit a center-to-periphery decreasing distribution, with the highest density in the central urban area and few in outer suburban areas such as Chongming. (3) A citywide space syntax analysis was conducted using the complete Shanghai road network, identifying 104 key planning grids from among 519 priority deployment grids. Taking Jiading District—which has the highest number of priority deployment grids (168)—as a case study, the space syntax analysis was used to narrow the selection down to 34 key planning grids.

**Conclusion:**

The three-tiered, progressive site selection framework developed in this study integrates macro-level predictions of functional suitability with micro-level assessments of road network accessibility. It provides urban planning authorities with actionable spatial decision-making references for identifying priority deployment areas for older adult(s) care facilities, and offers a replicable methodological approach for the planning of public service facilities in other high-density cities.

## 1 Introduction

Currently, population aging is a matter of common concern to the international community. According to a United Nations report, the global population aged 65 and older has reached approximately 857 million and is projected to approach 1.58 billion by 2050, accounting for 16% of the world's population (1). This aging trend is particularly pronounced in developing countries, primarily due to declining fertility rates and extended life expectancy. As the world's most populous developing country, China is experiencing a continuous growth in its older adult population and a deepening of societal aging (2). According to the National Bureau of Statistics of China (3), the country is projected to become a “super-aged society” by 2030, with the older adult population (aged 65 and above) exceeding 20%. At the same time, China's total population has been declining for several consecutive years. In 2025, the number of births stood at 7.92 million, a decrease of 1.62 million from the previous year, with the birth rate falling to 5.63‰—a historic low (4). The continuing acceleration of population aging and the sustained decline in the birth rate have led to a sharp rise in societal demand for older adult(s) care facilities (5).

Without scientific preliminary planning, issues such as inappropriate site selection and uneven spatial distribution will arise, which in turn will affect the quality of life for older adults, exacerbate regional disparities in resources, and hinder the achievement of the goal of universal coverage of older adult(s) care services (6). The “14th Five-Year Plan for National Economic and Social Development” issued by the State Council clearly outlines requirements for the development of the older adult(s) care service system, calling for the scientific planning of land use for new older adult(s) care facilities and prioritizing the utilization of existing facilities (7). The spatial distribution of older adult(s) care facilities directly affects service accessibility for older adults (8). Improper site selection not only results in a waste of resources but may also lead to some older adults being unable to access basic care services due to geographical isolation (9). Therefore, scientifically planning the siting of older adult(s) care facilities and optimizing their overall layout has become a critical issue in the field of urban planning (10). The national policy objective is to establish a “15-min older adult(s) care service circle” (54), meaning that older adults can access a variety of older adult(s) care services within a 15-min walk. The scientific selection of sites for older adult(s) care facilities is a crucial pre-requisite for achieving this policy objective. The basic service radius of a single facility typically corresponds to a 5-min living zone (approximately 500 m), which serves as the fundamental spatial unit constituting the 15-min service circle (11). A rational facility layout helps reduce gaps in service coverage and provides older adults with convenient and accessible older adult(s) care services (6).

With the widespread application of POI data, machine learning algorithms, and space syntax in geographic information systems, research on the site selection of older adult(s) care facilities has gained new technical tools and methodological support (12). As a method in the field of machine learning that combines predictive capability with interpretability, decision tree algorithms have been widely applied in research on the site selection and spatial layout prediction of urban public facilities (13). However, existing studies still have significant shortcomings in terms of spatial accuracy, cross-algorithm validation, and the practicality of site selection, necessitating improvements through a more systematic methodological framework. Therefore, this study constructs a three-tiered site selection decision-making framework that sequentially integrates POI functional environment prediction, aging population-based secondary screening, and space syntax-based local choice verification. By progressively refining the site selection results from the macro-grid level to the street scale, the framework provides urban planners with actionable spatial decision-making references. It offers a generalizable methodological approach for the planning of older adult(s) care facilities in other high-density cities. Compared with existing research, this study advances the field in three aspects: (i) POI data are used to construct predictive models, with street-level aging data introduced as a secondary screening criterion to prioritize the identification of grids with high aging needs within functionally suitable areas; (ii) multi-algorithm benchmark comparisons and chi-square tests are incorporated to transform subjective threshold judgments into quantifiable statistical evidence, thereby enhancing methodological rigor; and (iii) local choice indicators derived from space syntax analysis are employed as a third-tier screening criterion, translating macro-level predictions into street-scale planning decisions.

## 2 Literature review and research framework

Older adult(s) care facilities are an integral part of urban public services. As the urban population ages at an accelerating rate, the planning and construction of these facilities has become a new challenge in urban planning (14). The distribution of older adult(s) care facilities must gradually align with urban d5evelopment and the needs of older adult(s) care facilities (15). Scholars have conducted extensive research on the distribution of older adult(s) care facilities (16).

The first category involves the allocation of land for older adult(s) care facilities in urban planning. Traditional planning for older adult(s) care facilities typically adopts the perspective of land supply within the context of the city's master plan (17). Studies on the demand for land for older adult(s) care facilities primarily consider factors such as the demand for land in areas surrounding the city among the older adults, as well as the alignment between the behavior of the older adults and the supply and demand for such land (18). The second category of studies aims to evaluate and optimize the allocation efficiency of existing older adult(s) care facilities (19, 20). Research topics include geographical and spatial factors influencing the balanced distribution of older adult(s) care facilities (21, 22), the distribution characteristics of such facilities and the relationship between facilities and residents (14, 23), facility accessibility (24, 25), and the spatial coordination and continuity of facilities (26). The third category of research focuses on modeling and forecasting the future scale and types of older adult(s) care facilities (12, 27), primarily based on local natural population growth rates (28, 29).

Current research on older adult(s) care facilities in Shanghai has primarily focused on the coupled assessment of the spatial layout of existing facilities and population growth projections (17). However, modeling and predicting the future spatial distribution of older adult(s) care facilities represents an important area of research (12). In particular, POI data, as an emerging type of urban data, have been widely applied in research on urban functions and land-use planning. Many studies have utilized POI data to analyze the distribution of urban public facilities (30–32). Given the vast amounts of urban data requiring analysis, machine learning has been widely applied in urban big data analytics (33, 34). Compared to traditional GIS-based spatial decision support systems and agent-based modeling—the former of which relies on structured rules and struggles to identify non-linear relationships (35), and the latter of which involves complex modeling and demands high-quality data—machine learning offers unique advantages in analyzing non-linear issues within big data (33, 34). By analyzing large volumes of urban data, machine learning algorithms can identify complex patterns within the data and support spatial decision-making (36). Machine learning methods are widely used to analyze correlations among urban elements (37, 38), with specific algorithms selected based on the type of urban data and the research question at hand.

In addition, research has begun to emerge that uses POI data and machine learning to assess the current state of older adult(s) care facility siting and predict future locations. Some scholars have employed machine learning algorithms to analyze the impact of land cover, terrain, and population density on the siting of older adult(s) care facilities (39). Other scholars have used POI data and machine learning algorithms to construct decision tree models for simulating the site selection of older adult(s) care facilities in cities such as Guangzhou, Wuhan, and Lanzhou (10, 40, 41). The results indicate that this method demonstrates high predictive accuracy and spatial fit in the prediction of older adult(s) care facility site selection.

The aforementioned studies have advanced research on the site selection of older adult(s) care facilities in three key areas: at the data level, the incorporation of POI data and census data has expanded the information base for site selection analysis; at the methodological level, the application of machine learning algorithms has improved the accuracy of site selection predictions and the ability to identify non-linear relationships; and at the spatial level, the use of GIS spatial analysis tools has enhanced the spatial accuracy of facility layout planning.

However, existing research has the following shortcomings: (1) Regarding research data. In contrast, existing studies have incorporated POI and census data; they have failed to include street-level aging rates and population size as independent screening dimensions in the site selection analysis, making it difficult to fully reflect spatial variations in regional aging needs. (2) In terms of research methods, while existing studies have adopted machine learning algorithms, they primarily focus on model prediction accuracy and lack an in-depth interpretation of decision tree rules, making it difficult to identify the key factors influencing the site selection of older adult(s) care facilities and their relative importance. Furthermore, most existing studies rely solely on the ID3 algorithm, lacking cross-algorithm validation and statistical significance tests, which makes it difficult to fully ensure the robustness of model results. (3) Regarding site selection priorities and differentiated planning recommendations, most existing studies focus on identifying suitable sites but lack a systematic ranking of site priorities, making it difficult to clearly define priority areas and construction sequences for new facilities. Furthermore, the site selection results of existing studies lack micro-level road network accessibility verification, and there is a shortage of differentiated planning recommendations tailored to central urban areas, inner suburban areas, and outer suburban areas, making it difficult to provide planning departments with directly actionable spatial decision-making references.

**Figure 1:**
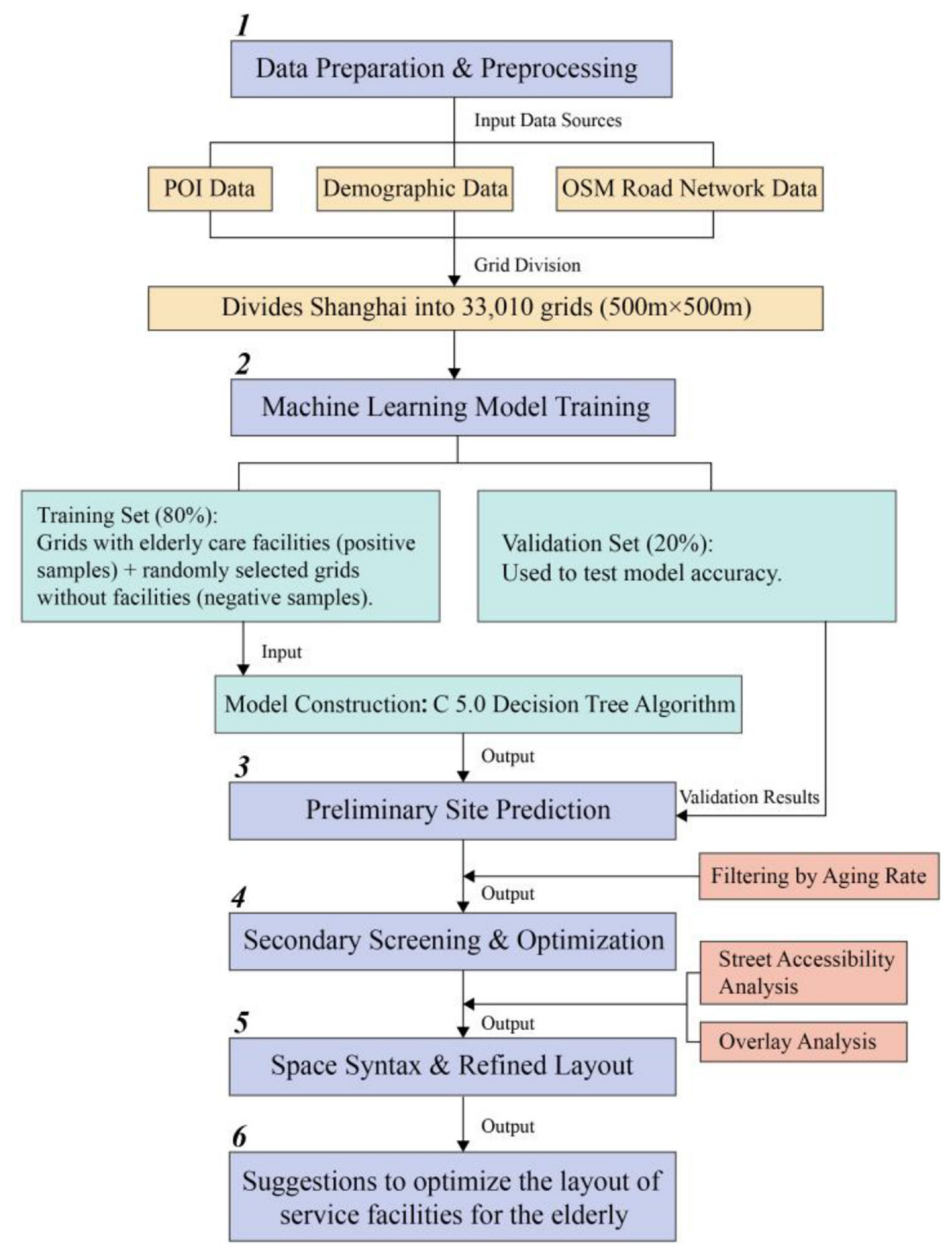
Methodological framework.

To address these shortcomings, this study builds upon existing POI and machine learning-based site selection research. It focuses on Shanghai to construct a three-tiered, progressive site selection framework to identify, in sequence, preliminarily suitable grids, priority deployment grids, and key planning grids. The specific research framework is shown in Figure 1.

This study consists of three main steps: (1) Data collection and processing. Based on their relevance to older adult(s) care facilities, the various types of urban facilities represented by POI data were categorized into 14 groups. Data on older adult(s) care facilities—including nursing homes, retirement homes, senior activity centers, senior apartments, and social welfare centers—were extracted from these groups and treated uniformly as the “older adult(s) care facilities” category for use as training samples. Using street-level aging data from the Seventh National Population Census, the city was divided into 33,010 grid cells. The spatial distribution characteristics of POIs within each grid were analyzed to construct a training dataset for site selection prediction. (2) Construction of a machine learning site selection model and multi-algorithm validation. Using the existing grid-level data of older adult(s) care facilities as the training dataset, a C5.0 decision tree site selection prediction model was trained. This model was then benchmarked against logistic regression and random forest algorithms to verify its reliability and robustness. The model's predictions were overlaid with the distribution of existing older adult(s) care facilities for validation. If the spatial match rate reached 80% or higher (42), the model was deemed to possess a certain level of spatial fitting capability. (3) Site selection prediction and secondary screening. First, the preliminary site selection results were presented, and a macro-level analysis was performed, comparing them with existing older adult(s) care facilities. Subsequently, secondary screening was conducted by combining the size and aging rate of the older adult population in each subdistrict to identify priority grid units. Finally, a space syntax-based local choice analysis was performed on the city's priority deployment grids using the complete Shanghai road network to identify key planning grids. Taking the district with the highest number of priority deployment grids as a case study, the refined process of space syntax screening was further demonstrated. Based on the differences among the central urban area, inner suburban areas, and outer suburban areas, differentiated recommendations for optimizing the layout of older adult(s) care facilities are proposed.

## 3 Study area

**Figure 2:**
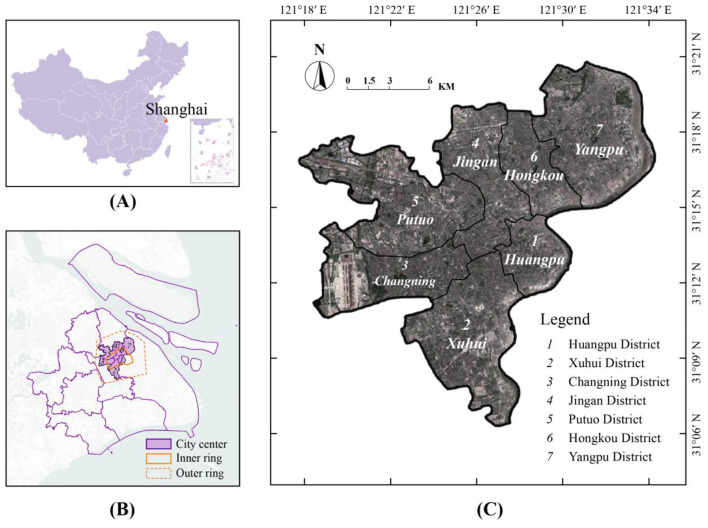
Location of the study area in Shanghai, China. **(A)** Location of Shanghai in China. **(B)** Spatial division of Shanghai into the central urban area, inner ring, and outer ring. **(C)** Location of the seven central urban districts examined in this study, including Huangpu, Xuhui, Changning, Jing'an, Putuo, Hongkou, and Yangpu.

Shanghai is situated in the southeastern coastal region of China, at the mouth of the Yangtze River, facing the Pacific Ocean (latitude 30°0′ to 31°3′, longitude 120°3′ to 122°2′; Figure 2). Covering approximately 6,340 km^2^, the city comprises 16 districts. As China's economic center and the most densely populated megacity, Shanghai is also one of the first cities in China to experience population aging, facing relatively concentrated population aging challenges (43). Its demographic characteristics are typical of a global metropolis. According to the General Urban Plan of Shanghai, the city is divided into three concentric spatial rings (44): the central urban areas (Xuhui, Yangpu, Jing'an, Huangpu, Hongkou, Changning, Putuo), inner suburban areas (Minhang, Baoshan, Jiading, Pudong New Area), and outer suburban areas (Chongming, Jinshan, Qingpu, Songjiang, Fengxian). Data from the (53) show that the older adult population aged 65 and above in Shanghai reached 4.05 million, accounting for 16.3% of the total population, indicating that Shanghai has entered an advanced stage of population aging (53). The aging population is growing rapidly but is unevenly distributed across regions, influencing the spatial adjustment and optimization of older adult(s) care facilities.

This study utilized administrative boundary data to divide Shanghai into 33,010 500 × 500 m grids (45), each assigned a unique identifier to facilitate further analysis of the spatial distribution of older adult(s) care facilities and other urban public service facilities. In this study, the 500 × 500 m grid serves primarily as the spatial analysis unit, consistent with similar studies on older adult(s) care facility site selection (10, 12, 40). It also corresponds to the service radius standard for older adult(s) care facilities within the “5-min living circle” as defined in the “Standards for Planning and Design of Urban Residential Areas (GB50180-2018).”

## 4 Data sources and processing

### 4.1 POI data

Roads, facilities, and buildings are the fundamental components of a city, and map data serves as the primary means of representing the urban landscape. POI data describes the spatial distribution of various functional zones within a city. The POI data used in this study were obtained via the Baidu Maps Developer API (https://lbs.baidu.com/, accessed November 20, 2025), and the program was written in Python. The acquired POI data are stored in a database format, containing facility names, functional categories, latitude and longitude coordinates, and other necessary attribute information. The POI data provided by the Baidu Maps API uses the BD-09 coordinate system. In this study, Python conversion code was used to transform the coordinates into the WGS 1984 geographic coordinate system, after which the data were imported into ArcGIS for spatial analysis. All spatial analyses in this study were conducted in ArcGIS 10.4 using the WGS 1984 UTM Zone 51N projection coordinate system.

### 4.2 Population data

The data on the older adult population in this study were derived from the Seventh National Population Census and were disaggregated to the level of 226 subdistricts. According to census data, there are 10 subdistricts in Shanghai with an older adult population (aged 65 and above) exceeding 40,000. Listed in descending order by population, they are: Dacang, Sanlin, Xinzhuang, Meilong, Yinxing, Gucun, Beicai, Chuansha, Jinyang, and Qibao. In addition, there are 81 subdistricts with an older adult population exceeding 20,000, accounting for 35.84% of all subdistricts; and 171 subdistricts with an older adult population exceeding 10,000, accounting for 75.66%.

**Figure 3:**
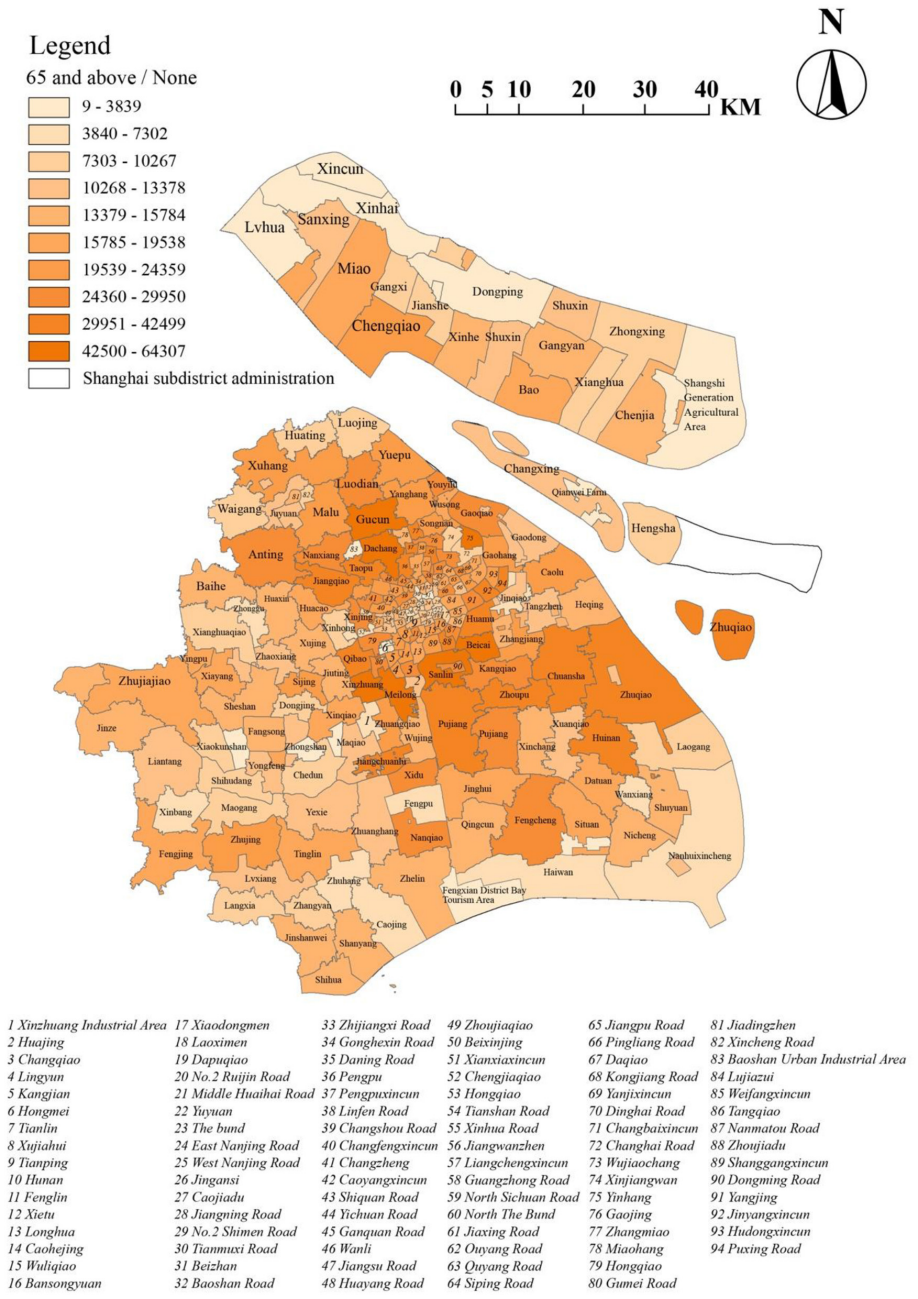
Distribution of the older adult population across subdistricts in Shanghai. (Coordinate system: WGS 1984 UTM Zone 51N).

**Figure 4:**
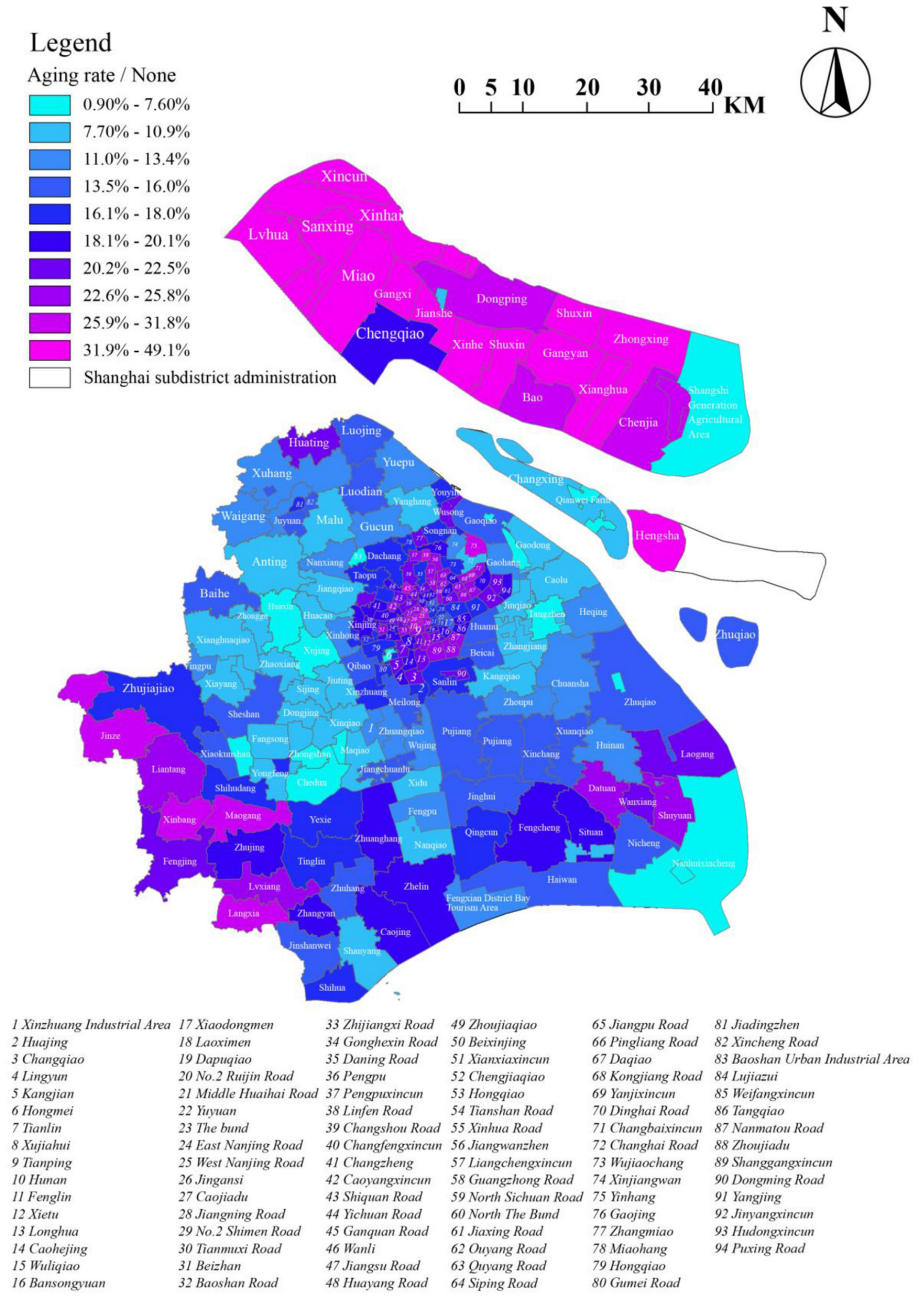
Distribution of population aging rate across subdistricts in Shanghai. (Coordinate system: WGS 1984 UTM Zone 51N).

The spatial distribution of Shanghai's older adult population shows a pattern of expansion from the central urban areas toward the outskirts, with growth clusters forming in some inner suburban areas in the north and east, before gradually declining toward the outer suburban areas (Figure 3). In terms of the aging rate, 215 subdistricts have an aging rate exceeding 7%. Of these, 77 subdistricts have an even higher aging rate, exceeding 20%, indicating that they have entered an advanced stage of population aging (Figure 4).

### 4.3 Infrastructure classification and meshing

Due to the large volume of raw POI data and the presence of duplicates or overlapping categories, pre-processing was required, including the removal of duplicate entries and points with missing attribute information (41).

Subsequently, considering the relevance to older adult(s) care facilities, the urban facilities represented by the POI data were classified into 14 categories (12): transport facilities, educational facilities, sports and leisure, attractions, life services, medical services, financial institutions, accommodations, shopping, catering, governmental institutions, companies, real estate, and older adult(s) care facilities. After classification, a total of 1,069,886 records were obtained (Table 1). From these, 1,670 records related to older adult(s) care facilities were selected. In this study, older adult(s) care facilities refer to institutions that provide care and social support services to older adults, including nursing homes, retirement homes, senior activity centers, senior apartments, and social welfare centers.

**Table 1:** Categories and quantities of POI related to service facilities for older adults.

Category I	Category II	Appellation of facilities	Number of POIs
Infrastructure services	Transport facilities	Airports, train stations, long-distance bus stations, subway stations, bus stops, etc.	78,745
	Education facilities	Libraries, science and technology museums, colleges and universities, primary and secondary schools, kindergartens, parent-child education, special education schools, training institutions, etc.	43,749
	Sports and leisure	Stadiums, fitness centers, farmhouses, cinemas, theaters, dance halls, leisure plazas, etc.	28,697
	Attractions	Parks, zoos, botanical gardens, museums, cultural relics, churches, scenic spots, etc.	4,727
	Life services	Communication offices, post offices, laundromats, photo studios, repair shops, newspapers and magazines, public restrooms, etc.	148,198
	Medical services	General hospitals, specialty hospitals, clinics, pharmacies, nursing homes, emergency centers, CDCs, etc.	21,560
Commercial services	Financial institutions	Banks, ATMs, credit unions, etc.	20,628
	Accommodations	Star hotels, express hotels, apartment hotels, etc.	19,175
	Shopping	Shopping centers, department stores, supermarkets, convenience stores, home building materials, home appliances, digital appliances, bazaars, etc.	270,348
	Catering	Chinese restaurants, foreign restaurants, snack and fast food stores, cake and dessert stores, cafes, cafeterias, etc.	129,579
Administrative office facilities	Governmental institutions	All levels of government, administrative units, welfare organizations, etc.	36,825
	Companies	Companies, parks, agriculture, forestry, and horticulture	194,540
	Real estate	Office buildings, residential areas, dormitories, etc.	71,445
Older adult(s) care facilities	Older adult(s) care facilities	Nursing homes, older adult(s) care centers, senior centers, older adult(s) apartments, social welfare centers, etc.	1,670
Total			1,069,886

**Figure 5:**
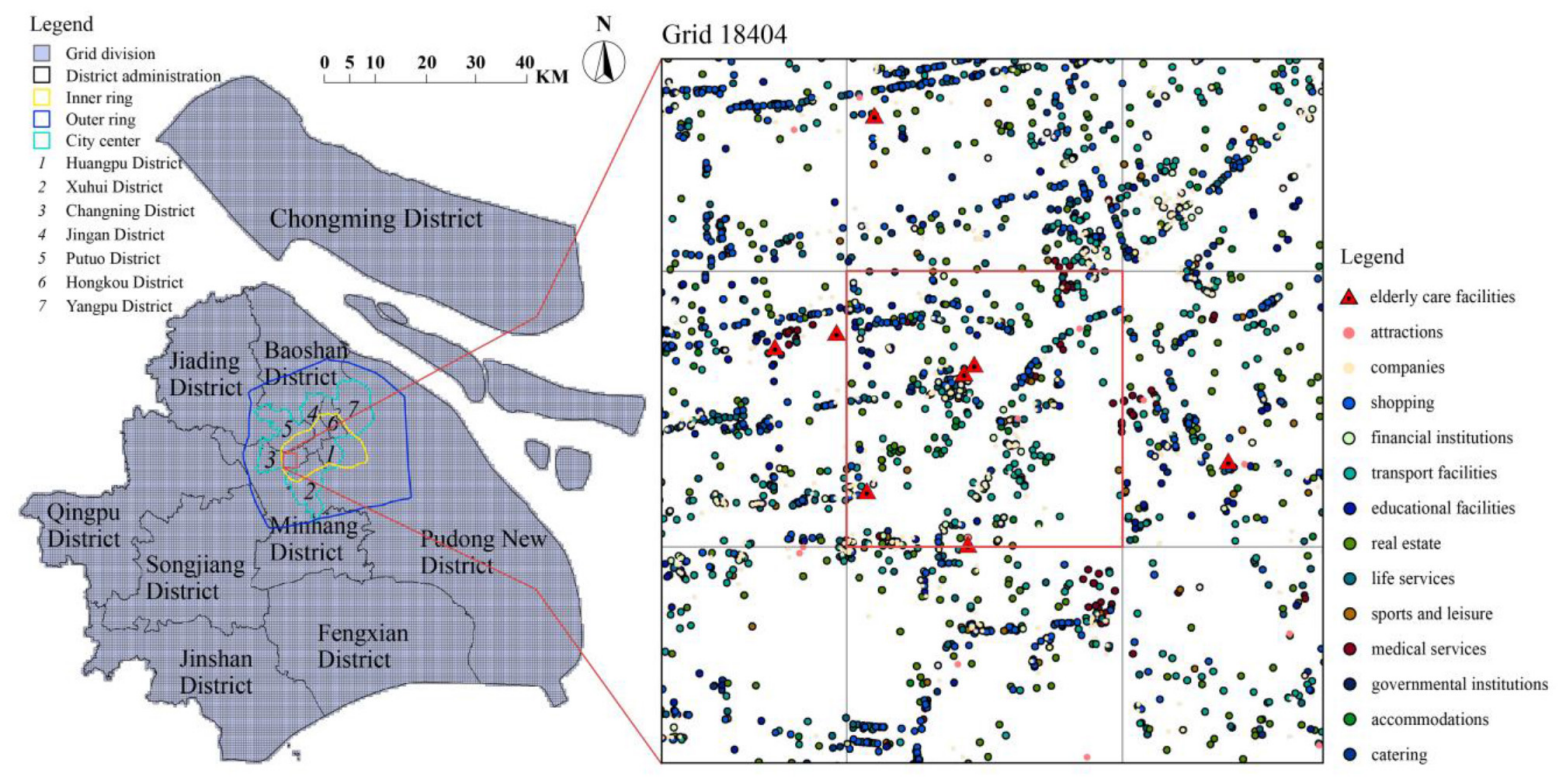
Spatial grid division of urban facilities in Shanghai and enlarged detail. (Coordinate system: WGS 1984 UTM Zone 51N).

This study divided Shanghai into 33,010 grid cells using a 500 × 500 m grid. Each grid cell was assigned a unique ID to facilitate subsequent analysis of the spatial distribution of various facilities. For example, Grid 18,404 in Changning District contains Older adult(s) care facilities such as the Dongting Home for Retired Cadres, Shanghai Jinfu Second Nursing Home, and Huayang Community Comprehensive Older adult(s) Service Center, as well as various other urban facilities, including transportation, sports and leisure, companies, healthcare, education, and catering (Figure 5). By analyzing the spatial distribution across all grids, this study determined whether specific types of facilities are present within each grid.

## 5 Research methodology

### 5.1 Grid layout and sample construction

First, the study area was divided into 500 × 500 m urban grids, with 500 m serving as the reference service radius for older adult(s) care facilities. Using the spatial overlay function in ArcGIS 10.4, POI location data were overlaid onto each grid to determine the distribution of urban public service facilities within each grid.

The criteria for determining whether each grid is suitable for the construction of older adult(s) care facilities are as follows: using association tools, all grids were linked, matched, and classified with the facilities. If a grid contains an older adult(s) care facility, indicating that this grid was selected as a facility location in the existing distribution, it was classified as a positive sample (assigned a value of +1); otherwise, it was classified as a negative sample (assigned a value of −1), thereby achieving a binary classification of potential sites for older adult(s) care facilities.

Subsequently, a number of grids equal to the number of positive samples was randomly selected from the negative samples. A 1:1 positive-to-negative sample ratio was adopted to avoid the impact of class imbalance on model training (10). The above grids collectively form a machine learning dataset, totaling 2,296 grids (1,148 positive samples and 1,148 negative samples). Eighty percent of the grids were randomly selected from this dataset to fit the model, while the remaining 20% were designated as the validation set to evaluate model accuracy (40). Of the 33,010 grids across the city, the 30,714 grids not used as training samples were designated as the final prediction dataset, thereby avoiding data overlap between the training samples and the prediction dataset.

### 5.2 C5.0 decision tree algorithm and multi-algorithm comparison

The C5.0 algorithm, developed by Quinlan as an improvement over C4.5, is a widely used method for constructing decision trees (46). The algorithm is known for its simplicity, ease of understanding, computational efficiency, and ability to effectively handle multi-valued attributes; it uses information gain ratio as its splitting criterion to recursively select the optimal splitting attribute at each node, thereby constructing a decision tree model. To verify the reliability and robustness of the C5.0 model, this study also incorporates logistic regression and random forests as benchmark models. Logistic regression is a classic linear classification method that offers good global interpretability; random forests improve predictive accuracy through a voting mechanism that aggregates multiple decision trees, effectively capturing non-linear interactions among variables (47). The three algorithms were trained and validated independently on the same dataset, and their predictive performance was evaluated through a comparative analysis to ensure the robustness and reliability of the final site selection results. The following are the key steps for constructing a decision tree model using the C5.0 algorithm.

Let the sample set be S = {S1, S_2_, …, S_n_} and the decision attribute set be D = {D1, D_2_, …, D_m_}. If the jth attribute Dj (j = 1, 2, …, m) has |Dj| samples, then the information entropy of D, info (D), is:


$$\begin{array}{l} \begin{array}{c} info\left(D\right)=-\sum_{j\;=\;1}^{m}p_{j}\backslash log_{2}p_{j} \end{array} \end{array}$$ (1)

Here, pj represents the probability of class j, which is typically estimated as the proportion of samples in that class relative to the total number of samples. If the set of values for attribute A is {A1, A_2_, …, A_k_}, and there are |A_i_| samples under the i-th value A_*i*_ (i = 1, 2, …, k), where |A_ij_|denotes the number of samples in A_i_ that belong to class j, then the information entropy after partitioning by attribute A is given in Equation 2:


$$\begin{array}{l} \begin{array}{c} info\left(A\right)=-\sum_{i\;=\;1}^{k}\frac{\left|A_{j}\right|}{|S|\sum_{j\;=\;1}^{m}p_{ij}\backslash log_{2}p_{ij}} \end{array} \end{array}$$ (2)

The information gain before and after splitting attribute A is:


$$\begin{array}{l} \begin{array}{c} Gain\left(A\right)=info\left(D\right)-info\left(A\right) \end{array} \end{array}$$ (3)

Building on Equations 1–3, the C5.0 algorithm introduces SplitInfo(A) to normalize the information gain. The formula for SplitInfo(A) is shown in Equation 4:


$$\begin{array}{l} \begin{array}{c} SplitInfo\left(A\right)=-\sum_{i\;=\;1}^{k}\frac{\frac{\left|A_{i}\right|}{|S|\backslash {log}_{2}\left(\left|A_{i}\right|\right)}}{\left|S\right|} \end{array} \end{array}$$ (4)

The information gain ratio, GainRatio (A), is calculated using Equation 5:


$$\begin{array}{l} \begin{array}{c} GainRatio\left(A\right)=\frac{Gain\left(A\right)}{SplitInfo\left(A\right)} \end{array} \end{array}$$ (5)

The C5.0 algorithm calculates the information gain ratio for each attribute before and after splitting. At each step, it selects the attribute with the highest information gain ratio as the basis for splitting the current node, thereby using the optimal classification attribute as the specific classification criterion. Through repeated iterations, the algorithm ultimately constructs a decision tree model for identifying priority deployment areas for older adult(s) care facilities in Shanghai.

### 5.3 Site selection prediction and secondary screening

Grid data, excluding the 2,296 training sample grids, were input into the pre-trained site selection model for older adult(s) care facilities to generate a preliminary prediction of the overall distribution of older adult(s) care facilities in Shanghai and identify preliminarily suitable grids. To verify the statistical significance of the spatial match rate, this study employed a random baseline hypothesis and chi-square tests to statistically validate the prediction results, with a significance level set at *P* < 0.001. This study also utilized an independent validation set (comprising 20% of the training samples) to evaluate model accuracy, ensuring the reliability of the prediction results.

Given the large number of preliminary prediction results, it was necessary to conduct a secondary screening in conjunction with aging population data to identify priority deployment areas with relatively high aging demand. Based on data from the Seventh National Population Census, this study applied dual screening criteria: an aging rate exceeding 7% [the internationally recognized standard for an aging society (40)] and an older adult population exceeding 17,000 (the median older adult population size across Shanghai's 226 subdistricts based on the Seventh National Population Census). Subdistricts meeting these criteria were identified as priority development areas. The specific procedure was as follows: using the ArcGIS 10.4 spatial join tool, the preliminary suitable grids were spatially overlaid with the administrative boundaries of eligible subdistricts to extract grids located within the subdistrict boundaries; subsequently, grids already containing existing older adult(s) care facilities were excluded, ultimately identifying the priority deployment grids.

### 5.4 Space syntax analysis

Space syntax is a method for analyzing spatial configurations (48). As space syntax has evolved, researchers have discovered that certain indicators correlate with human spatial behavior; consequently, these indicators are used to predict the potential impact of urban spaces on users. Within the space syntax framework, space is composed of various elements, which can be represented as maps and graphs, and connectivity and integration are visualized through network analysis.

This study employs DepthmapX as the analytical tool, an open-source, cross-platform spatial analysis tool suitable for spatial networks at various scales (49), ranging from individual buildings to urban districts. Axis maps were constructed in DepthmapX based on OSM road network data, with axes drawn according to the principle of the longest line of sight to cover all accessible streets, followed by graphical analysis of the generated network. This study calculates local choice indicators. While global integration reflects the overall accessibility of streets within the entire urban network, local choice focuses on micro-connectivity within a 500-m radius (11), which aligns with the actual walking capabilities of older adults.

First, a local choice analysis was conducted on the city's priority deployment grids—selected through a secondary screening process—using the complete Shanghai road network. Referencing the top 20% high-accessibility threshold widely adopted in similar space syntax studies (42, 49), grids with low accessibility were excluded to identify key planning grids across the city. Subsequently, the district with the highest number of priority deployment grids after the second screening was selected as a case study. A spatial overlay analysis was conducted between the district's priority deployment grids and the space syntax axis map to further illustrate the refined process of space syntax screening. To eliminate boundary effects in the case study analysis, the road network scope was expanded to include a 10-km buffer zone beyond the administrative boundaries of the case study area.

## 6 Results

### 6.1 Multi-algorithm benchmark comparison

Table 2 presents a benchmark comparison of different machine learning models on the dataset used in this study. The results show that the C5.0 decision tree model achieved the highest prediction accuracy (83.36%) on the independent validation set, outperforming logistic regression (82.05%) and random forests (80.96%). The C5.0 decision tree model performed best across all four metrics—accuracy, precision, recall, and F1 score—demonstrating its relative advantage in capturing the complex spatial logic of older adult(s) care facility site selection.

**Table 2:** Benchmark comparison of machine learning models.

Model	Accuracy	Precision	Recall	F1-score
Decision tree (C5.0)	83.36%	84.88%	80.31%	82.52%
Random forest	80.96%	84.49%	73.76%	78.71%
Logistic regression	82.05%	85.63%	75.07%	79.95%

### 6.2 Decision tree model results and key factor identification

The core process of site selection for older adult(s) care facilities involves continuously defining requirements for the surrounding spatial environment and progressively eliminating spaces that do not meet these criteria. The presence or absence of various types of facilities in the site selection process corresponds to the various splitting nodes in a decision tree model. In contrast, the final determination of whether a grid cell is suitable for older adult(s) care facilities corresponds to the classification result at the leaf nodes of the decision tree.

**Figure 6:**
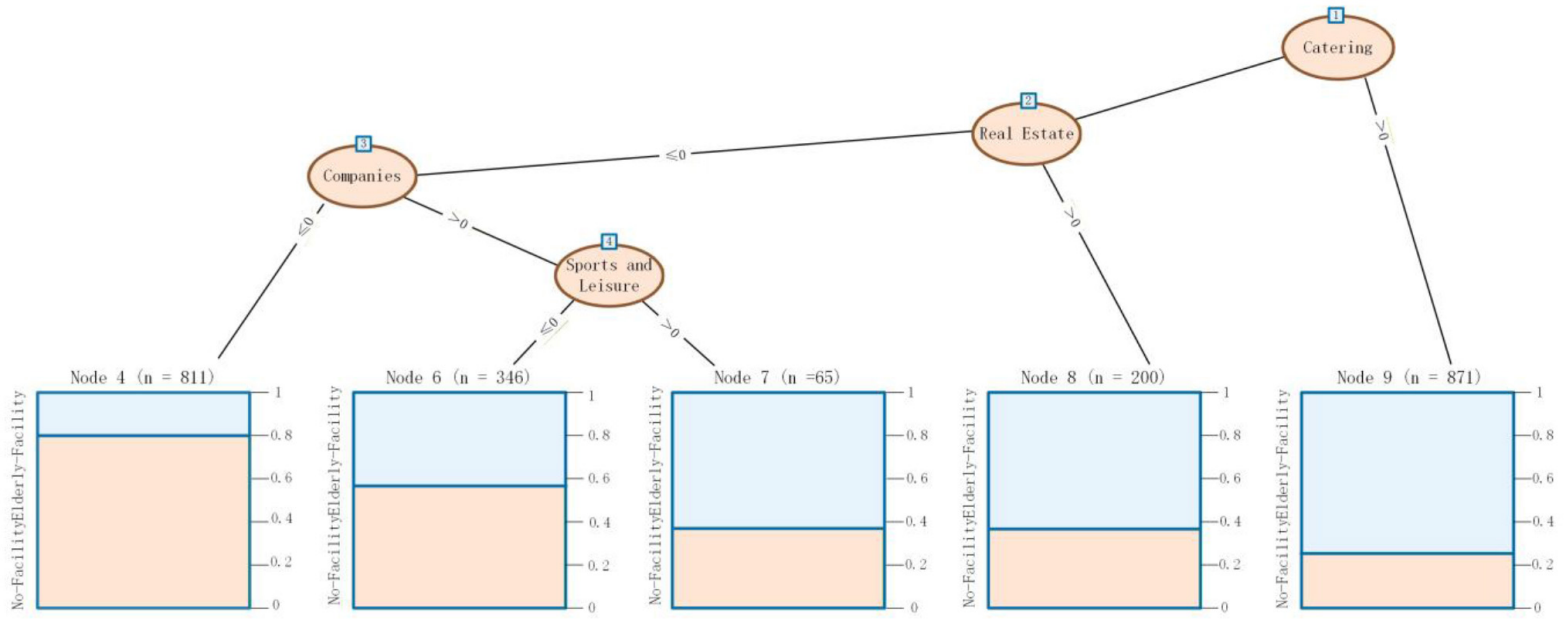
C5.0 decision tree analysis for older adult(s) care facility siting.

This study used the counts of 13 types of POI facilities within each grid as independent variables and the presence of older adult(s) care facilities within the grid as the dependent variable. A decision tree was trained using the C5.0 algorithm, resulting in a decision tree model for the site selection of older adult(s) care facilities in Shanghai (Figure 6). In Figure 6, each leaf node is represented as a bar chart, where the blue portion indicates the proportion of grids within that node that contain existing older adult(s) care facilities, and the orange portion represents the proportion of grids that do not contain older adult(s) care facilities. The higher the blue proportion, the more likely it is that the grids meeting the corresponding splitting criteria are suitable for the construction of older adult(s) care facilities. The first four layers of the decision tree sequentially feature four categories of POI facilities: catering, real estate, companies, and sports and leisure. The left and right branches are denoted by ≤ 0 and >0, respectively, indicating the presence or absence of such facilities within the grid. According to the principles of the C5.0 algorithm, attributes closer to the upper layers of the decision tree exert a more significant influence on site selection decisions.

The first level is catering, indicating that it has the most significant influence on the site selection of older adult(s) care facilities in Shanghai. When catering facilities are present in a grid cell (>0), the process proceeds directly to the leaf node (Node 9, *n* = 871). This node has a blue proportion of 0.76, the highest among all nodes, indicating that grid cells containing catering facilities have the highest suitability for older adult(s) care facilities; when catering facilities are absent (≤0), the process proceeds to the second level for further evaluation.

The second layer is real estate facilities. In grids lacking catering facilities, real estate further distinguishes site suitability. Grids with real estate facilities (>0) proceed to the leaf node (Node 8, *n* = 200), where the blue proportion is 0.62, indicating high suitability; grids without real estate facilities (≤0) proceed to the third layer for further evaluation.

The third level consists of companies. In grids lacking both catering and real estate facilities, the presence of companies further distinguishes site suitability. Grids with no companies (≤0) proceed to the leaf node (Node 4, *n* = 811), where the blue proportion is only approximately 0.20—the lowest among all nodes—indicating the lowest suitability for older adult(s) care facilities in such grids; grids with companies (>0) proceed to the fourth level for further evaluation.

The fourth layer consists of sports and leisure facilities. In grids lacking catering and real estate facilities but containing companies, the presence of sports and leisure facilities further refines the suitability of the site. Grids with sports and leisure facilities (>0) enter the leaf node (Node 7, *n* = 65), where the proportion of blue is approximately 0.62, indicating a certain spatial correlation between the presence of sports and leisure facilities and higher suitability for site selection; when they are absent (≤0), they enter the leaf node (Node 6, *n* = 346), where the proportion of blue is approximately 0.42.

### 6.3 Preliminary site selection prediction results

Based on the aforementioned training model, a suitability assessment was conducted on all 33,010 grids across Shanghai, identifying a total of 940 preliminarily suitable grids for older adult(s) care facilities. Using the existing 1,148 older adult(s) care facilities as a spatial validation reference, the results showed that 89% of the existing facilities (1,022 out of 1,148) matched the predicted results. This indicates that the model effectively captured the distribution patterns of the functional environments of existing facilities, providing a statistical basis for identifying potentially suitable areas.

To verify the statistical significance of this match rate, this study introduced a random baseline hypothesis: if the 1,148 existing older adult(s) care facilities were randomly distributed among the city's 33,010 grids, the probability that any given facility would fall within one of the 940 model-identified suitable grids would be only 2.85% (940/33,010). The chi-square test results (χ^2^ = 30,517, *p* < 0.001) indicate that the model's predictions exhibit a high degree of spatial non-randomness, suggesting that the 89% match rate reflects an objective correlation between the spatial characteristics of POIs and the distribution of older adult(s) care facilities, and is statistically significant.

**Figure 7:**
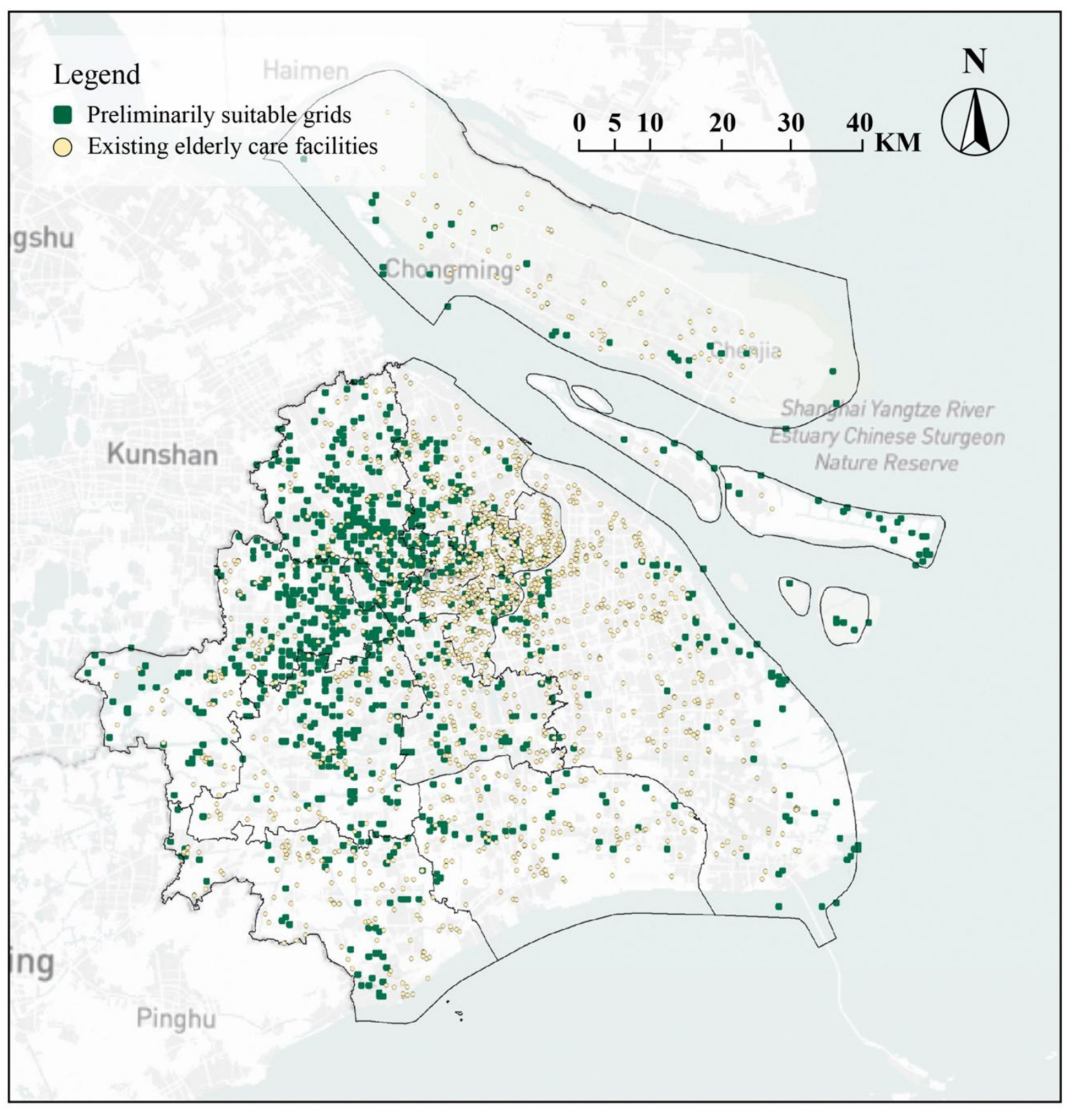
Preliminary prediction results of the layout of older adult(s) care facilities in Shanghai. (Coordinate system: WGS 1984 UTM Zone 51N).

Based on the preliminary prediction results (Figure 7), the spatial distribution of older adult(s) care facilities in Shanghai exhibits the following characteristics: existing facilities are primarily concentrated in the central urban areas and gradually decrease with increasing distance from the city center. Specifically, central urban districts such as Jing'an, Huangpu, Xuhui, Hongkou, and Yangpu have a relatively large number of existing older adult(s) care facilities; in contrast, inner suburban areas such as Jiading and Baoshan have fewer existing facilities, but preliminary predictions indicate a large number of suitable grids in these areas. In outer suburban areas such as Fengxian, Chongming, and Jinshan, preliminarily suitable grids are primarily concentrated in the central areas of each district. In contrast, the distribution of other grids is relatively scattered. On Hengsha Island in Chongming District, due to its unique geographical environment, preliminarily suitable grids are primarily concentrated near the Yangtze River estuary. There is a significant spatial correlation between preliminarily suitable grids outside the central urban areas and major transportation hubs (especially subway stations and highway interchanges). Among these, areas such as Nanxiang in Jiading and Huaxin in Qingpu have a relatively large number of preliminarily suitable grids.

### 6.4 Secondary screening based on population aging

Given the large number of 940 grids identified in the initial screening, it was necessary to further optimize the selection by incorporating data on the aging population. According to data from the Seventh National Population Census, the proportion of the population aged 65 and older in 215 subdistricts of Shanghai exceeded 7% of the total population, meeting the internationally recognized standard for an aging society. Based on this, 84 subdistricts with an older adult population exceeding 17,000 were further identified as priority development areas.

**Figure 8:**
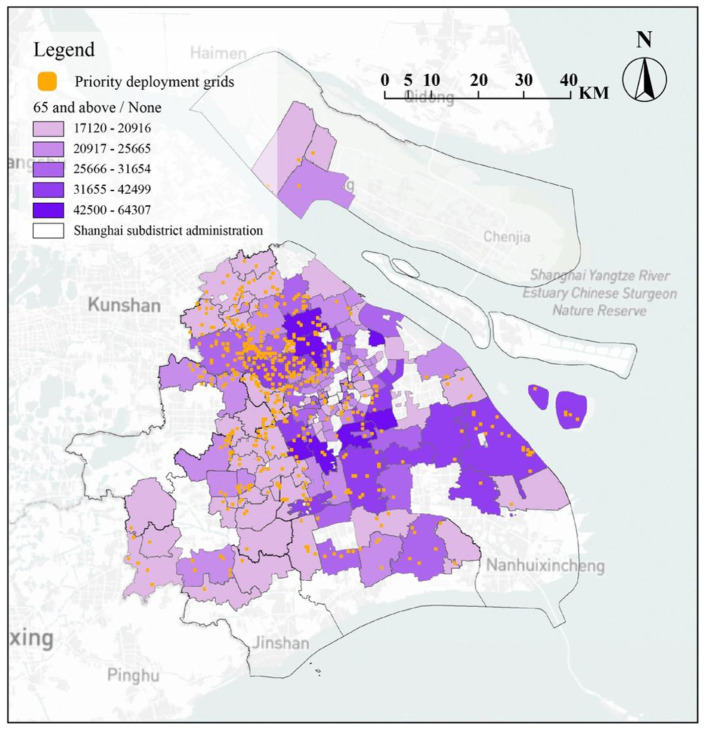
Distribution of priority deployment grids in Shanghai. (Coordinate system: WGS 1984 UTM Zone 51N).

Using ArcGIS 10.4, preliminary grids were identified within the administrative boundaries of the aforementioned subdistricts. After excluding grids with existing older adult(s) care facilities, a total of 519 priority deployment grids were ultimately identified (Figure 8). These priority deployment grids are primarily concentrated in the northwestern inner suburban areas and regions surrounding the Inner Ring Road, with high concentrations of older adult(s) residents, with the highest number of grids located in Jiading, Qingpu, and Minhang districts.

In terms of spatial distribution, the number of priority deployment grids decreases progressively from the inner city toward the outer rings, with the highest density in the central urban area, followed by the inner suburban areas. In contrast, outer suburban areas such as Chongming have very few priority deployment grids. Overall, the spatial pattern exhibits characteristics of core agglomeration and a decreasing trend across concentric rings.

Among the 215 subdistricts with an aging rate exceeding 7%, 77 have rates exceeding 20%. Sanxing Town in Chongming District has the highest aging rate in the city at 49%, yet it has very few priority deployment grids. Subdistricts with the largest older adult populations are primarily concentrated in Yangpu, Jing'an, and Hongkou districts around the Inner Ring Road, as well as in the southwestern part of Baoshan District and the central-western part of Pudong New Area.

Currently, 12 subdistricts in the inner suburban areas have reached super-aged levels, with 10 located in Pudong New Area and two in Baoshan District. It is worth noting that although the aging rates in the inner suburban areas are relatively low, the total older adult population remains substantial, particularly in areas adjacent to the central urban districts, such as Jinyang Xincun Subdistrict in Pudong New Area.

In the outer suburban areas, subdistricts with higher aging rates are primarily concentrated in border zones adjacent to surrounding townships and provincial boundaries, central subdistrict areas, and around transportation hubs connecting to the city center. Although 14 subdistricts in Chongming District have aging rates exceeding 30%, the total older adult population in these areas is significantly lower than the average for other subdistricts in Shanghai.

### 6.5 Space syntax analysis and key planning grid identification

**Figure 9:**
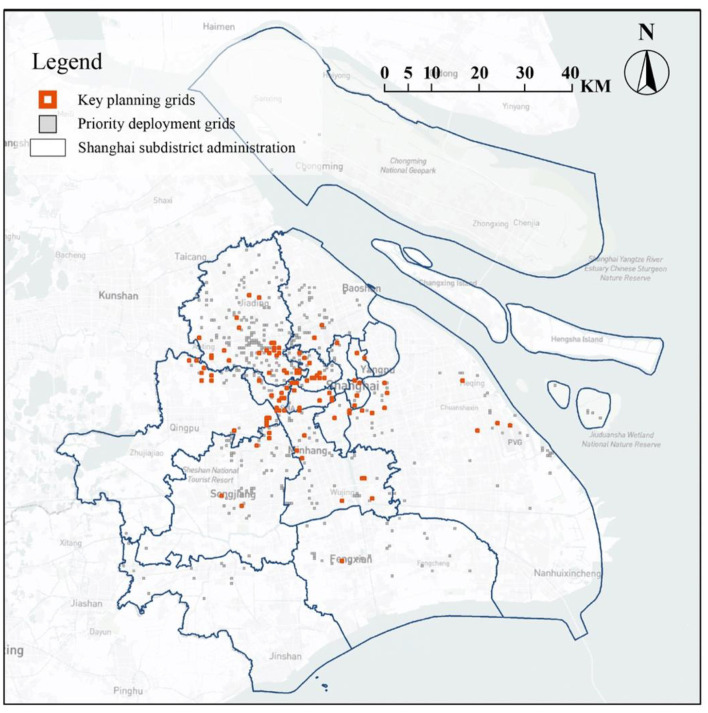
Key planning grids for older adult(s) care facilities in Shanghai. (Coordinate system: WGS 1984 UTM Zone 51N).

At the urban scale, this study applied a spatial overlay method to Shanghai's citywide road network and 519 priority deployment grids. By overlaying the 519 priority deployment grids with the top 20% of streets in terms of local choice, the study ultimately identified 104 key planning grids that meet both the requirements of functional suitability and road network accessibility (Figure 9).

The spatial distribution of the 104 grids exhibits a distinct “center-periphery” gradient. Most are concentrated in the central urban areas, including Jing'an, Putuo, Changning, Xuhui, Huangpu, and Yangpu districts, where street connectivity is high, and the older adult population is concentrated. Secondary clusters are distributed along the suburban transition zone, particularly evident in districts such as Minhang and Baoshan. In contrast, the number of key planning grids in the outer suburban areas (such as Qingpu, Songjiang, Jinshan, Fengxian, and Chongming) is relatively small, and they are primarily concentrated in the central areas of administrative towns rather than in dispersed rural areas.

**Figure 10:**
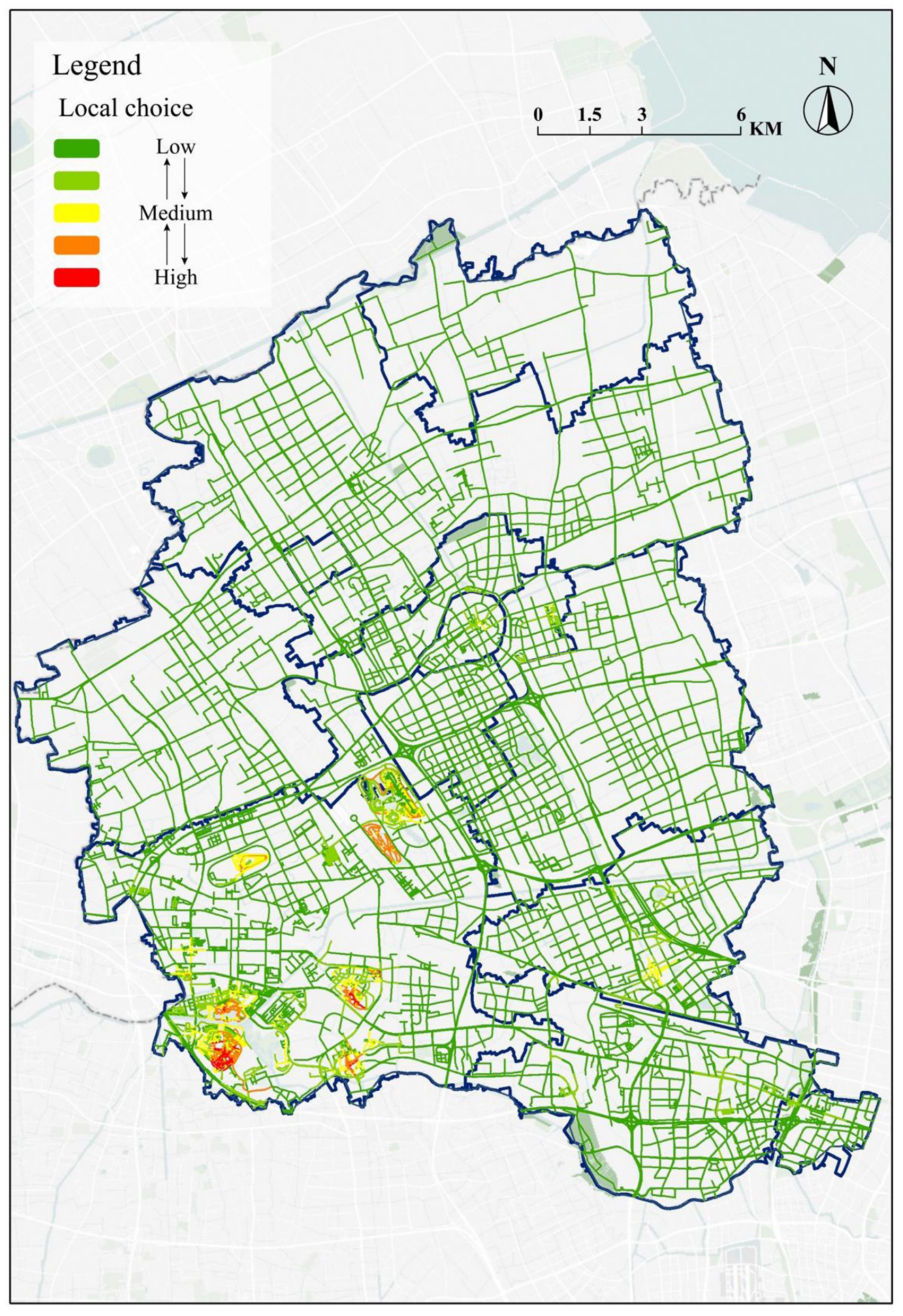
Local choice analysis map of Jiading District. (Coordinate system: WGS 1984 UTM Zone 51N).

**Figure 11:**
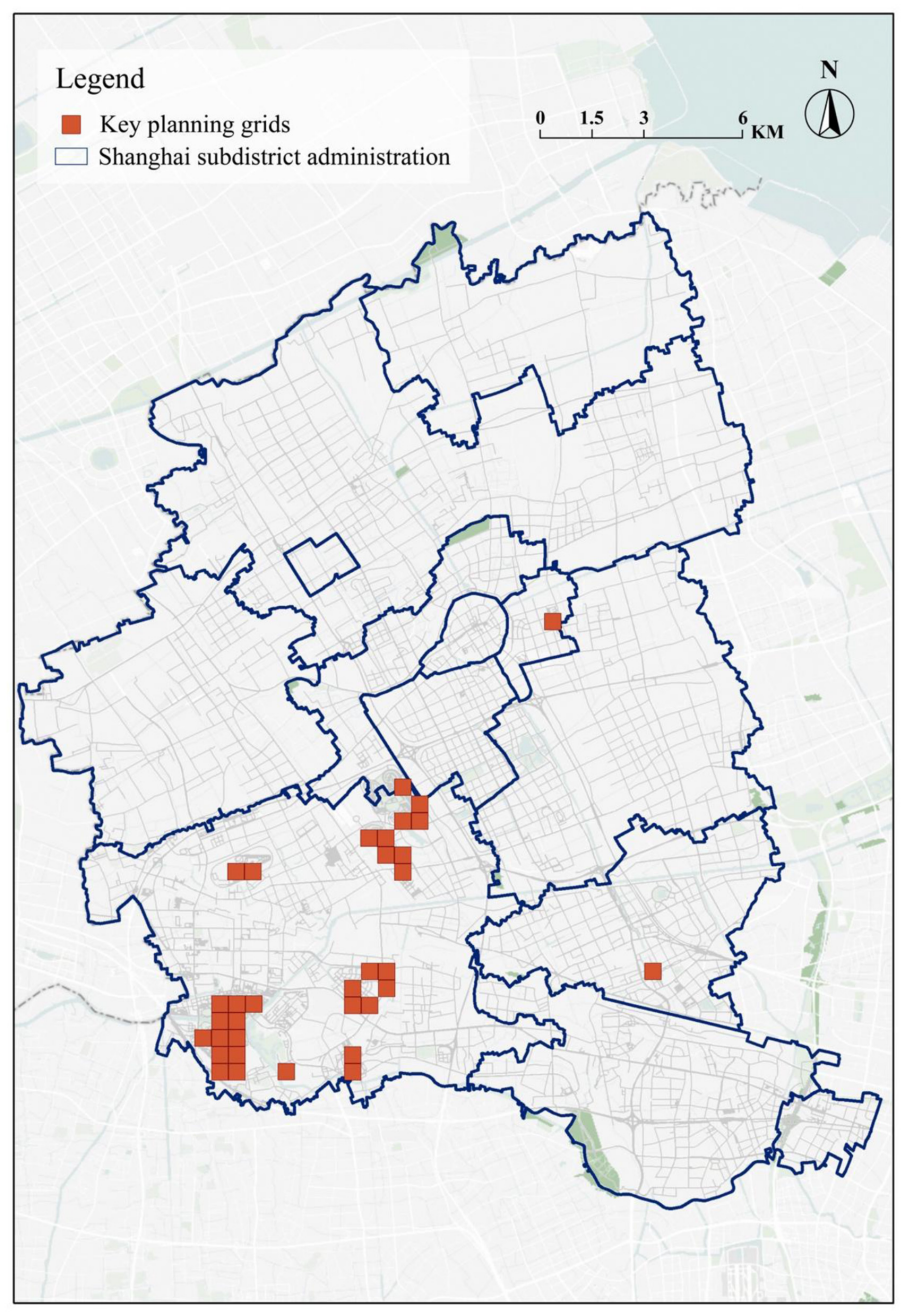
Key planning grids for older adult(s) care facilities in Jiading District. (Coordinate system: WGS 1984 UTM Zone 51N).

Subsequently, taking Jiading District as a typical case study, the study further illustrated the refined process of space syntax screening. Jiading District has the highest number of priority deployment grids for older adult(s) care facilities, totaling 168. Areas with high local choice values in Jiading District are primarily concentrated in the southwest, including the central areas of Anting Town and Jiading Town, as well as Nanxiang Town, which borders Putuo District (Figure 10). By spatially overlaying the top 20% of streets by local choice with the 168 priority deployment grids (Figure 11), a strong spatial consistency was observed between functional suitability and road network accessibility. This analysis ultimately identified 34 key planning grids, primarily located in the southwestern areas of Anting Town, the central areas of Jiading Town, and Nanxiang Town.

## 7 Discussion

This study integrates POI data, machine learning algorithms, and space syntax to construct a three-tiered progressive screening framework. Key findings indicate that catering, real estate, and company facilities are critical functional environmental factors for the siting of older adult(s) care facilities in Shanghai, reflecting a tendency for such facilities to cluster in areas with well-developed daily living services and high concentrations of residential and employment opportunities within high-density urban environments. The priority deployment grids exhibit a center-to-periphery decreasing distribution, reflecting gradational differences in functional environmental suitability across different concentric zones. The space syntax local choice verification translates macro-level functional suitability into street-scale planning decisions, effectively enhancing the planning feasibility of the site selection results.

Compared with traditional planning methods based on land supply or natural population growth rates, this study constructs a supply-side site selection model using POI data. It incorporates street-level aging data as a secondary screening criterion, thereby improving the accuracy of the site selection results in identifying areas with aging-related needs.

Regarding the factors influencing the location of older adult(s) care facilities, this study found that catering facilities, rather than medical facilities, are the strongest spatial predictor of such facility locations in Shanghai. This finding is unexpected, as existing research typically regards medical facilities as the primary factor (12, 42), whereas data from Shanghai indicates that catering facilities have greater predictive power. Areas with a rich catering environment tend to reflect the overall functional vitality of the surrounding area and often provide comprehensive daily living services, which may constitute a necessary condition for the operation of older adult(s) care facilities and the accessibility of services for older adults. This finding echoes a recent study on the distribution of community dining spaces for older adults in Beijing (50), and together they point to the same policy implication: the logic behind the siting of older adult(s) care facilities should shift from “proximity to medical care” to “daily living services.”

Real estate, as the second key influencing factor, indicates that older adult(s) care facilities tend to cluster in areas with high residential population density, consistent with the basic site selection logic that such facilities should be located close to the people they serve (40). Companies, as the third key influencing factor, reflect the spatial pattern whereby older adult(s) care facilities tend to cluster in areas with high employment density. This pattern is particularly pronounced in the context of Shanghai's high-density urban environment, where employment-dense areas typically feature mixed-use developments and comprehensive daily service amenities, exhibiting a strong spatial correlation with the clustered distribution of older adult(s) care facilities. This differs from the findings of the Xi'an study (42), where government agencies and medical facilities were identified as the dominant factors. As a historic and cultural city, Xi'an's community service system is dominated by these institutional facilities. In contrast, as a highly commercialized megacity, Shanghai's mixed-use developments have made daily life service facilities—such as catering facilities—a more prevalent feature of the built environment.

Regarding model accuracy and cross-city generalizability, this study achieved an 89% spatial match rate with existing older adult(s) care facilities, consistent with the results of similar studies in cities such as Guangzhou and Wuhan (10, 40). This cross-city consistency may reflect the spatial similarities of mixed-use development patterns driven by China's rapid urbanization process, indicating that POI-based machine learning site selection methods possess a certain degree of methodological transferability in the context of China's high-density megacities. Unlike previous studies, which primarily employed the ID3 algorithm and used a fixed 80% threshold as the sole validation criterion (40, 42), this study employed the C5.0 algorithm alongside logistic regression and random forests for independent multi-algorithm validation, with the accuracy rates of the three algorithms proving highly consistent. Furthermore, the introduction of a random baseline hypothesis and chi-square tests confirmed the statistical significance of the spatial match rate, ruling out the possibility of random distribution. These two measures collectively enhanced the robustness and rigor of the method validation.

During the site selection phase, existing studies in this field primarily rely on the concentration of the older adult population in each subdistrict for secondary screening (10, 40). Furthermore, these studies are limited to predicting functional suitability at the grid level and do not incorporate road network accessibility verification, making it difficult to provide planning departments with actionable planning recommendations. This study introduces the space syntax local choice indicator as a third-tier screening criterion, combining macro-level functional suitability with micro-level road network accessibility. At the citywide scale, 104 key planning grids were identified from 519 priority deployment grids through space syntax screening. Taking Jiading District as a case study, the 168 priority deployment grids were further refined to 34 key planning grids. The analysis results at both scales indicate that incorporating road network accessibility verification on top of functional suitability can further refine the site selection results to the street level, providing planning departments with more actionable spatial references. The POI data and census data required for this framework are readily available in major Chinese cities. Both the C5.0 decision tree and space syntax tools are open-source or publicly accessible, and do not rely on specific municipal administrative data, enabling direct application to the site selection planning of public service facilities in other high-density cities.

Regarding the center-to-periphery decreasing concentration pattern in spatial distribution, the high concentration of older adult(s) care facilities in Shanghai's core area and the resulting concentric decline closely align with the spatial distribution patterns shaped by historical land accumulation, the inertia of administrative resource allocation, and population density gradients. The central urban area has formed a concentration of existing infrastructure based on historical land advantages. In contrast, the density of older adult(s) care facilities in the inner suburban and outer suburban areas is relatively low. This spatially decreasing pattern aligns with the distribution pattern of “central concentration and peripheral sparsity” commonly observed in China's urban public service facilities (51), reflecting the ongoing influence of historical development paths on the spatial layout of older adult(s) care facilities. Further analysis, combined with aging data, reveals spatial disparities between the distribution of facilities across different concentric zones and the needs of the older adult population. This disparity is most pronounced in Sanxing Town, Chongming District, where a 49% aging rate coexists with a relatively limited supply of older adult(s) care facilities. As a rural area with low POI density, the case of Sanxing Town demonstrates that the spatial distribution of older adult(s) care facilities does not always align with the geographic distribution of the older adult population's needs, a phenomenon particularly evident in outer suburban areas with relatively low POI density.

Based on the identification results derived from the three-tier screening framework described above, this study proposes the following differentiated spatial optimization recommendations for the three zones. The central urban area has the highest facility saturation but faces the most severe shortage of new land; therefore, optimization strategies should focus on utilizing existing resources. It is recommended to use incentive policies, such as tax breaks, to convert idle commercial or industrial buildings into community-level older adult(s) care facilities, while tailoring layout plans to the unique characteristics of each community. For example, in areas with significant gaps, such as Putuo District, the density of small-scale, community-embedded older adult(s) care facilities should be increased. In contrast, in commercial centers like Huangpu District, the development of large-scale, vertically integrated older adult(s) care facilities should be explored. Inner suburban areas have relatively abundant land resources, particularly in towns with significant population growth, such as Nanxiang in Jiading and Huaxin in Qingpu. In these areas, land should be reserved at the urban-rural fringe for the construction of medium- to large-scale older adult(s) care facilities, and a transit-oriented development model should be adopted to integrate these facilities with rail transit hubs. In outer suburban areas, a multi-center cluster model centered on town-level administrative centers can be adopted. Medical, nursing, rehabilitation, and recreational facilities should be concentrated in areas rich in ecological resources. Priority should be given to improving transportation accessibility in regions with high aging rates but poor road network connectivity, thereby preventing older adults from falling into a service vacuum due to geographical isolation. These recommendations for differentiated planning aim to provide spatial references for planning authorities across different zones, ensuring that older adults can access necessary services within a reasonable service radius. This approach reduces the burden of travel, strengthens a sense of community belonging, and enhances overall wellbeing.

Beyond macro-level strategies, micro-level accessibility within the road network is also a key factor influencing the construction of older adult(s) care facilities. The introduction of space syntax addresses the scale limitations of traditional grid-based predictions. A case study of Jiading District shows that while some grids are identified as suitable sites at the macro level due to their abundance of POIs, they exhibit low local choice values at the space syntax and road network topology levels. Therefore, the introduction of local choice indicators adds analytical depth to POI-based predictions, making the site selection results better reflect actual planning conditions at the street scale. However, this study also reveals a noteworthy issue in the site selection of older adult(s) care facilities: the trade-off between high physical accessibility and a quiet environment. While highly accessible streets certainly facilitate the daily mobility of older adults and help them integrate into community life, the noise and air pollution associated with high-traffic thoroughfares may adversely affect the environmental quality of older adult(s) care facilities (52). Therefore, this study recommends incorporating both accessibility and environmental quality into site selection criteria through inward-facing spatial design and multi-level vertical green buffers.

Although this study has achieved certain results, the following limitations remain.

First, as this study is based on urban POI data, it is suitable for cities that are relatively mature and have comprehensive data coverage. For new urban districts or areas where infrastructure development is not yet complete, POI data may be incomplete, leading to some bias in the analysis results. Furthermore, POI data cannot reflect the scale, capacity, and service quality of existing older adult(s) care facilities, nor does it incorporate physical and economic constraints such as land use planning, land prices, and rental costs. Future research could further integrate multi-source data, such as land use classifications and economic indicators, to construct a more comprehensive site selection predictionframework.

Second, this study's machine learning model uses grids containing older adult(s) care facilities as positive samples to learn supply-side location patterns based on POI functional and environmental characteristics; this approach has certain limitations. Some grids that are suitable for development but have not yet been developed may be mislabeled as negative samples, limiting the model's ability to identify undeveloped but potentially suitable areas. The introduction of aging data as a secondary screening criterion fails to fully cover areas with high demand but sparse POI environments during the prediction phase. Furthermore, the importance of key factors such as catering facilities and companies may partly reflect the background effect of the overall high density of POIs in urban built-up areas; the spatial associations identified by the model reflect statistical correlations rather than causal relationships. Future research could further validate the independent effects of each factor by controlling for the overall POI density variable and introducing methods such as geographically weighted regression, and explore the development of a dual-driver site selection prediction framework that incorporates both supply-side functional environmental characteristics and demand-side demographic characteristics, so as to more comprehensively identify priority development areas with high demand but scarce facilities.

Third, this study did not categorize older adult(s) care facilities by type or grade, instead incorporating different types of facilities into the model training as a single category. Since different types of facilities vary in terms of site selection logic and functional environmental requirements, this mixed training may cause the model to capture an average of the site selection logic across different facility types, thereby reducing the predictive value of the results for specific planning decisions to some extent. Future research could develop separate site selection prediction models for each facility type to enhance the practical applicability of the findings for planning purposes.

Fourth, the 20% threshold used in the space syntax screening stage was based on standard practices in similar studies; however, the optimal threshold for different urban contexts remains to be further validated. Furthermore, the conclusions of this study are closely tied to the specific urban development context of Shanghai, as the logic behind the siting of older adult(s) care facilities varies significantly depending on urban density, land use regulations, and models of older adult(s) care services. Future research could conduct a systematic comparison of different threshold settings through sensitivity analysis and extend this methodology to other urban contexts to further validate the robustness and generalizability of the results.

## 8 Conclusion

Focusing on Shanghai as the study area, this research developed a three-tiered, progressive site selection framework that integrates POI data, machine learning algorithms, and space syntax. The main conclusions are as follows:

(1) The results of the independent validation of the three algorithms were highly consistent (C5.0: 83.36%; Logistic Regression: 82.05%; Random Forest: 80.96%), demonstrating the cross-algorithm robustness of the predictions. A chi-square test (χ^2^ = 30,517, *p* < 0.001) further confirmed the statistical significance of the 89% spatial match rate. The decision tree hierarchy revealed that catering facilities are the strongest predictor of older adult(s) care facility siting in Shanghai, followed by real estate and companies, indicating that older adult(s) care facilities tend to cluster in urban built-up areas with mature functional environments.

(2) Using aging rates and older adult population size as dual screening criteria, 519 priority deployment grids were identified from 940 initially suitable grids. Spatial analysis shows that priority deployment grids are highly concentrated around the Inner Ring Road and in the northwestern inner suburban areas, with the highest number of grids in Jiading, Qingpu, and Minhang districts. In contrast, grids are extremely scarce in outer suburban areas due to the scarcity of POIs.

(3) A citywide space syntax analysis reduced the 519 priority deployment grids to 104 key planning grids, primarily concentrated in the central urban area and the inner suburban transitional zone. In the outer suburban areas, grids are mainly concentrated in the central areas of administrative towns. Taking Jiading District—which has the highest number of priority deployment grids (168)—as a case study, space syntax screening reduced this to 34 key planning grids, primarily located in areas with high road network accessibility in the southwest, such as Anting Town, Jiading Town, and Nanxiang Town. The consistency between results at the two scales demonstrates that road network accessibility verification effectively translates macro-level functional suitability into street-level planning decisions.

(4) The data and tools required for the multi-level site selection framework developed in this study are publicly available and do not rely on specific municipal administrative data, making it directly applicable to the site selection planning of public service facilities in other high-density cities.

## Data Availability

The original contributions presented in the study are included in the article/supplementary material, further inquiries can be directed to the corresponding author.
